# Association between Physical Activity and Non-Alcoholic Fatty Liver Disease in Adults with Metabolic Syndrome: The FLIPAN Study

**DOI:** 10.3390/nu14051063

**Published:** 2022-03-03

**Authors:** Catalina M. Mascaró, Cristina Bouzas, Sofia Montemayor, Miguel Casares, Cristina Gómez, Lucía Ugarriza, Pere-Antoni Borràs, José Alfredo Martínez, Josep A. Tur

**Affiliations:** 1 Research Group on Community Nutrition and Oxidative Stress, University of the Balearic Islands, 07122 Palma de Mallorca, Spain; c.mascaro@uib.es (C.M.M.); cristina.bouzas@uib.es (C.B.); sofiamf16@gmail.com (S.M.); cristina.gomez@ssib.es (C.G.); luciaugarriza@gmail.com (L.U.); 2 Health Institute of the Balearic Islands (IDISBA), 07120 Palma de Mallorca, Spain; 3 CIBEROBN (Physiopathology of Obesity and Nutrition CB12/03/30038), Instituto de Salud Carlos III (ISCIII), 28029 Madrid, Spain; 4 Radiodiagnosis Service, Red Asistencial Juaneda, 07011 Palma de Mallorca, Spain; casaresmiguel@gmail.com; 5 Clinical Analysis Service, University Hospital Son Espases, 07120 Palma de Mallorca, Spain; 6 Camp Redó Primary Health Care Center, 07010 Palma de Mallorca, Spain; 7 Department of Pedagogy and Specific Didactics, University of the Balearic Islands, 07122 Palma de Mallorca, Spain; pa-borras@uib.es; 8 Cardiometabolics Precision Nutrition Program, IMDEA Food, CEI UAM-CSIC, 28049 Madrid, Spain; jalfredo.martinez@imdea.org

**Keywords:** physical activity, Mediterranean diet, Mediterranean lifestyle, metabolic syndrome, non-alcoholic fatty liver disease

## Abstract

Background: A lifestyle with regular PA (physical activity) and Mediterranean diet has benefits on NAFLD (non-alcoholic fatty liver disease) and MetS (metabolic syndrome). Objectives: To assess the association between physical activity and NAFLD in adults with MetS. Design: Cross-sectional study in 155 participants (40–60 years old) from Balearic Islands and Navarra (Spain) with diagnosis of NAFLD and MetS, and BMI (body mass index) between 27 and 40 Kg/m^2^. Methods: PA level was categorized into two groups according to weekly METs (metabolic equivalents of tasks). PA was assessed using a validated Minnesota questionnaire and accelerometers. MetS parameters were assessed by blood collection analysis and NAFLD by abdominal MRI (magnetic resonance imaging). Results: Participants with high PA showed more energy expenditure and expended more calories than ingested (−143.9 Kcal/day; *p* < 0.001). PA was a risk factor for AST (aspartate aminotransferase) (adjusted OR: 7.26; 95% CI: 1.79–29.40) and a protective factor for ALT (alanine aminotransferase) (adjusted OR: 0.24; 95% CI: 0.12–0.48), GGT (gamma-glutamyl transferase) (adjusted OR: 0.52; 95% CI: 0.29–0.94) and IFC-NMR (intrahepatic fat content by nuclear magnetic resonance) (adjusted OR: 0.26; 95% CI: 0.12–0.56) when sociodemographic confounders were considered. Conclusions: NAFLD patients with high PA showed more positive relationship on MetS parameters and liver profile (ALT, GGT, IFC-NMR) than subjects with low PA, but not for AST. Difference between calories ingested and expended influenced this relationship.

## 1. Introduction

Non-alcoholic fatty liver disease (NAFLD) is the most common liver disease, with an overall prevalence higher than 25% [1]. It is characterized by steatosis in 5% or more of the hepatocytes, without being caused by excessive alcohol consumption and/or drug-induced steatosis [2]. It can be influenced by different risk factors, including age, metabolic syndrome (MetS), insulin resistance, sociodemographic characteristics, diet, and physical activity (PA) [2,3]. Most of them can be modified by lifestyle, so it is important for NAFLD prevention and reversion [1].

NAFLD risk increases with age and MetS [4]. The main parameters that constitute MetS are obesity, hypertension, insulin resistance, and dyslipidemia, which are directly related to NAFLD [5]. Some characteristics influencing NAFLD are body mass index (BMI), age, gender, smoking habit, alcohol consumption, and low educational level [6]. NAFLD is influenced by diet, so diet is usually considered as a treatment for NAFLD [7]. This is the case of the Mediterranean diet, defined by a low intake of carbohydrates (especially sugars and refined sugars) and by a high intake of omega-3 and monounsaturated fatty acids. Moreover, the Mediterranean lifestyle includes the practice of regular PA. In this way, it has been pointed out that an association between physical activity and risk of nonalcoholic fatty liver disease [8,9], and people with NAFLD showed low levels of PA [6].

Active lifestyle with regular PA has proven health benefits. It decreases high plasma triglyceride levels, weight, and blood pressure, as well as regulates low plasma high-density lipoprotein (HDL)-cholesterol levels. It improves type 2 diabetes mellitus and MetS [10].

There is no specific and successful pharmacological treatment for NAFLD. Healthy lifestyle with regular PA and diet are the best present treatments [11]. Most of the existing literature reported diet effects on NAFLD, but the relationship between PA and NAFLD is still scarce. Therefore, the aim of the current study was to assess the association between PA and NAFLD in adults with MetS.

## 2. Materials and Methods

### 2.1. Design, Setting, and Ethics

**Figure 1:**
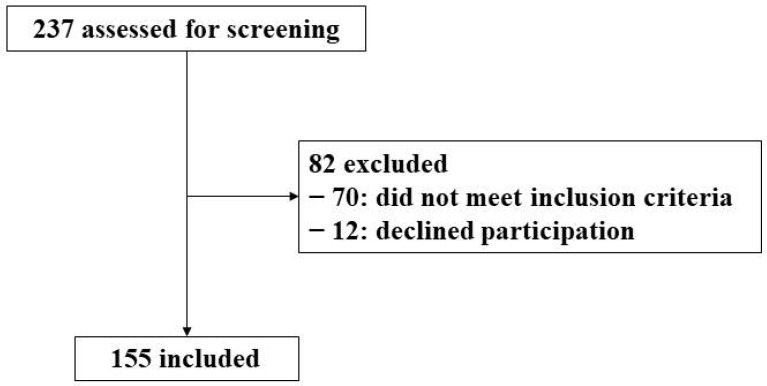
Study flow-chart.

The current study involves 155 participants aged 40–60 years, with NAFLD diagnosed by magnetic resonance imaging (MRI; Signa Explorer 1.5T, General Electric Healthcare, Chicago, IL, USA), with BMI 27–40 kg/m^2^, and meeting at least three MetS criteria as described by the International Diabetes Federation (IDF) [12]. Exclusion criteria were previous cardiovascular disease, congestive heart failure, liver diseases (other than NAFLD), cancer or a history of malignancy in the previous 5 years, previous bariatric surgery, acute febrile illnesses, urinary tract infections, post-renal hematuria, hemochromatosis, protein overload, non-medicated depression or anxiety, alcohol and drug abuse, pregnancy, primary endocrinological diseases (other than hypothyroidism and type 2 diabetes mellitus), concomitant therapy with steroids, in-tense physical exercise, or being unable to provide informed consent. In the first session, participants were asked for their stable body weight and stable calorie intake in the six months previous to their inclusion in the trial. If they answered negatively, then they were admitted to the trial. Screening and selection for inclusion are shown in the study flow-chart (Figure 1).

The study protocol and procedures were performed following the Declaration of Helsinki ethical standards and approved by the Ethics Committee of Research of Balearic Islands (ref. IB 2251/14 PI). The study was registered at ClinicalTrials.gov number NCT04442620 (https://clinicaltrials.gov/ct2/show/NCT04442620) (accessed on 25 February 2022) [13]. All participants were informed about the study and they provided their written informed consent prior to participation.

### 2.2. Anthropometrics, Dietary Intake, and Fitness and Physical Activity

Anthropometric measurements were taken (weight, height, BMI, waist, hip, and neck circumferences) as described elsewhere [14]. Dietary intake was measured by means of a validated food frequency questionnaire (FFQ) [15]. Energy and nutrient intakes were calculated as frequency multiplied by nutrient composition of specified portion size for each food item, using a computer program based on available information in Spanish food composition tables [16,17].

Physical activity was measured by accelerometry (ActiGraph wGT3X-B; ActiGraph LLC, Pensacola, FL, USA). Mean weekly time of PA was also collected, in minutes, using the validated Spanish version of Minnesota Leisure Time Physical Activity Questionnaire. The reported energy expenditure was interpreted as metabolic equivalents of task (MET)·minute/week [18,19].

### 2.3. NAFLD Diagnosis

For liver imaging, participants had to undergo a magnetic resonance imaging (MRI) to confirm the diagnosis of NAFLD and to know the amount of liver fat (Signa Explorer 1.5T, General Electric Healthcare, Chicago, IL, USA) [20].

### 2.4. Blood Collections Analysis

Blood samples were collected from antecubital vein after 12 h overnight fast. Biochemical analysis included fasting plasma glucose, liver transferases (alanine aminotransferase (ALT), aspartate aminotransferase (AST) and gamma-glutamyl transferase (GGT)), triglycerides, and high-density lipoprotein (HDL) cholesterol. They were quantified by standard enzymatic methods, as previously described [14].

### 2.5. Other Health Outcomes

Lifestyle and socioeconomic data were collected (age at inclusion, gender, civil status, education level, socioeconomic status according to job, smoking habit, and alcohol consumption). Socioeconomic status according to job was classified as low, medium, and high based on their own occupation and of the head of household, if applicable [21]. Smoking habit was grouped in binary categories, non-smoker, or smoker (≥1 cigarette/day) [22]. Moreover, hepatic steatosis index (HSI) (no/yes > 36) and fatty liver index (FLI) (no/yes > 60) were calculated [23]. Alcohol consumption was classified as 0 alcoholic drinks, <7 drinks/week and ≥7 drinks/week without becoming alcoholic [20]. Blood pressure was measured in triplicate using a validated semi-automatic oscillometer (Omron HEM-705CP, Omron Healthcare Inc., Lake Forest, IL, USA) as previously described [14].

### 2.6. Statistics

Analyses were performed with the SPSS statistical software package version 27.0 (SPSS Inc., Chicago, IL, USA). Data are shown as mean, standard deviation (SD) and median, interquartile range (IQR). Differences among groups were tested with by Mann–Whitney U-test since all parameters analyzed were non-normally distributed. Prevalence is expressed in sample size and percentage. Difference in prevalence among groups was tested using chi-square test (all *p* values were two-tailed).

Multivariate analysis was used. Level of PA was catalogued according to 50th percentile (P50). MetS criteria (dependent variables) were associated to two PA groups: high (>P50 of MET/day) and low (<P50 of MET/day), as independent variables, and markers of fatty liver, as dependent variables, were associated to PA groups (high and low) (independent variables). In the first case, five criteria of MetS, for each item, three odds ratio (OR) were calculated: crude, adjusted by sociodemographic factors (age and gender), and adjusted by both sociodemographic factors and difference between ingested and expended calories. In the second case, indicators of fatty liver, for each item, four OR were calculated: crude, adjusted by health factors (elevated fasting glucose, elevated triglycerides, and hypertension), sociodemographic factors (age, gender, smoking habit, alcohol consumption, and ingested–expended calories), and both adjusted by health and sociodemographic factors.

Cut-off points for the variables for which OR has been performed were a value of waist circumference ≥94 cm in men and ≥80 cm in women was considered abdominal obesity; fasting glycemia ≥ 100 mg/dL, triglycerides ≥ 150 mg/dL, and high-density lipoprotein cholesterol < 40 mg/dL in men and <50 mg/dL in women as indicators of MetS; systolic pressure ≥ 130 mmHg and/or diastolic pressure ≥ 85 mmHg as hypertension [24]. Serum levels of AST > 40 U/L, ALT > 40 U/L, and GGT > 50 U/L in men and >32 U/L in women were considered clinically important [25]. A mean percentage of liver fat ≥ 6.4% was considered clinically important too for intrahepatic fat contents by nuclear magnetic resonance (IFC-NMR) [26].

Results were considered statistically significant if *p*-value < 0.05.

## 3. Results

Table 1 shows sociodemographic characteristics according to PA levels (high PA: >P50; low PA: <P50). Participants with higher PA level were younger, had slightly higher weight and hip circumference, and lower neck circumference. They had higher protein intake (113.6 g/day), in contrast to a lower lipid intake (93.9 g/day); having a higher caloric expenditure (2515.3 Kcal/day: expended calories in the table). The difference between ingested and expended calories was negative in the high PA group (−143.9 Kcal/day), confirming higher calorie expenditure than calorie intake. Regarding energy expenditure, subjects with high PA also had high energy expenditure, both accelerometer-measured and self-reported METs (2.1 and 0.4 MET/day, respectively). The high PA group had a difference between measured and reported MET higher than the low PA group (1.9 MET/day). In the low PA group, there were more men than women, while in the high PA group the proportion of men and women remained similar. Most of the subjects included in the low PA group did not smoke (94.9%), while in the high PA group, up to a 17.5% of participants were smokers.

Health characteristics according to PA levels are available in Table 2. High plasma levels of AST, ALT, and GGT were more likely to be found among participants in the low PA group. Moreover, subjects in the low PA group had a higher percentage of IFC-NMR than subjects in the high PA group. The high PA group had a lower percentage of participants with ALT, FLI, and IFC-NMR values above the clinically relevant threshold (indicators of NAFLD) compared to the low PA group. The same was observed for triglycerides, blood pressure and fasting blood glucose (indicators of MetS).

Table 3 shows crude and adjusted OR for association between MetS parameters and PA levels. Low PA was established as the reference. OR crude and adjusted-1 analysis showed that PA acted as a protective factor in front of high triglycerides and high fasting glycemia. PA seemed to be a protective factor for hypertension but after adjustment for age and weight, significance was lost. When difference in calories was considered (OR adjusted-2), PA was not related to all MetS parameters.

Lastly, crude and adjusted OR for association between indicators of NAFLD and PA levels are shown in Table 4. Low PA was established as the reference. After adjustment by confounding variables, higher levels of PA were considered as a risk factor for high AST. However, high PA was a strong protective factor for ALT, which was not modified by either health or sociodemographic characteristics. After adjustment for sociodemographic confounding variables, PA became protective for GGT, and also protective for IFC-NMR.

## 4. Discussion

Physical activity is a protective factor for triglyceridemia and glycemia in NAFLD patients. Previous studies reported that a healthy lifestyle, based on the Mediterranean diet and regular PA, decreased these plasma levels in both NAFLD [27,28] and without NAFLD [29].

Previous studies showed that PA promotion programs were successfully decreasing hypertension [30,31]. However, current results showed that, after adjustment by confounders, PA was not significant for blood pressure.

Increased energy expenditure (due to regular PA) and low caloric intake in adults with NAFLD and MetS improved the MetS parameters analyzed in the current study [20]. Since PA and ingested-to-expended calories ratio were closely related, PA influenced MetS parameters but not after adjustment by the ratio of ingested-to-expended calories.

AST is a liver enzyme whose monitoring allows knowing the state of liver damage [32]. Previous literature showed that it is usual to find high AST levels in people with NAFLD and MetS, without other chronic liver diseases [33]. It is also usual that people who practiced regular PA also increased AST levels as a marker of muscular damage [34]. It could explain why high PA is a risk factor for AST after adjustment by sociodemographic characteristics.

In the current study, PA was a protective factor for ALT, GGT, and IFC-NMR. ALT and GGT are two liver enzymes which variation shows liver damage [32]. High PA increased ALT serum levels, but lower than AST levels. However, few hours after PA ended, ALT levels returned to baseline [34].

Improvement of ALT levels was associated with a notable reduction of hepatic fat contents, which means an improvement of NAFLD [35]. An intervention with Mediterranean diet and PA sessions of moderate intensity in NAFLD patients, allowed a decrease in weight, BMI, and waist circumference. Moreover, they improved MetS parameters and normalized some liver enzymes, especially ALT [27,28,36]. This explains why the current results showed that high PA was a strong protective factor for ALT in NAFLD patients.

Weight loss and histological improvement of the liver damage was associated with the decrease of GGT levels [35]. As it has already seen, a Mediterranean lifestyle, following a low-calorie Mediterranean diet and regular PA, was associated with weight loss and improvement of MetS parameters in NAFLD patients [10]. Therefore, it confirms the current hypothesis that higher PA, considering the sociodemographic characteristics as confounders, may act as a protective factor against high GGT levels in NAFLD patients.

Supporting the current results about the protective factor of PA for IFC-NMR, previous studies reported that exercise and PA training decreased intrahepatic fat contents in NAFLD patients, but also in obese people without this disease. Both interventions contemplated a healthy lifestyle together with a correct diet to achieve this effect [37,38]. Health and sociodemographic characteristics also played an important role in this effect because, so far, lifestyle intervention was the best treatment for NAFLD [37].

Previous evidence showed that low PA is more common in people with excess of weight [39]. This is contrary to the current findings in which participants with high PA had slightly higher weight, perhaps because these data were collected at the beginning of the study and no intervention was applied yet. A similar situation was found in the anthropometric parameters, which can be strong predictors of NAFLD and liver fat contents [40]. People with higher PA showed a remarkable improvement in anthropometric parameters, because PA contributed to decrease weight and BMI [41]. In comparison to the current results, this was only observed for neck circumference, which was lower in the high PA group from the start of the study. However, hip circumference was higher in the high PA group from the starting point, so it would be necessary to assess this evolution over time.

Previous literature reported that an increased protein intake, along with PA, was related to a decrease of body fat, weight, and energy intake in overweight and obese adults. Previous research showed that PA with enough protein intake from a healthy diet (such as the Mediterranean diet) had beneficial effects on body composition and reduction of intrahepatic fat in NAFLD patients [42,43]. All these conclusions agree with the current findings (the high PA group had higher protein intake). Regarding lipids, the current high PA group had lower lipid intake. A previous study showed that Mediterranean diet or low carbohydrate diet were more efficient than low fats diets. Sometimes, quality was more important than the amount of ingested fats [44]. Accordingly, the type of fat in the Mediterranean diet was considered in the current study.

Regular PA increased energy expenditure or caloric expenditure. Therefore, people with high PA expended more calories than people with low PA. This fact, together with a balanced diet, resulted in a clear weight loss [45,46]. In this regard, the current study found that the high PA group had more expended calories than the low PA group, which means a negative difference between ingested and expended calories.

High PA and energy expenditure were beneficial to decrease intrahepatic fat contents and risk of NAFLD [47]. In the current study, energy expenditure was reported through two ways, one measured with accelerometers and other reported by participants through the Minnesota questionnaire. Both ways measured energy expenditure with MET and showed an increase of daily energy expenditure in high PA individuals. The difference between measured and reported energy expenditure was positive and higher in the high PA group, notifying that people in this group reported spending less than they really did. As the existing literature pointed out, it is usual that higher PA has higher energy expenditure. Moreover, there is often a weak correlation between the METs reported by PA questionnaires and those measured by accelerometry, and it is common to report higher PA than that measured with the accelerometer [48], despite current findings it is the other way around. This could be because it is difficult to remember or accurately estimate all the activities performed or their intensity in case of PA questionnaires. In contrast, accelerometers assess PA objectively and continuously [49].

## 5. Strengths and Limitations

The main strength of this study is that it analyzed several aspects of the same sample and at the same time-point: biochemical, dietary, PA, and liver imaging data. In addition, liver imaging is performed by MRI, one of the most sensitive and accurate techniques to quantify the intrahepatic fat contents [50]. Other strength of the current study is the use of two different methods to assess PA: questionnaire and accelerometer. On the top of that, used methods could be easily transferred into clinical practice to treat NAFLD.

The main limitation was the small sample size. Another important limitation is that causal inferences cannot be established. The simultaneous measurement does not allow knowing the time sequence of events. Lastly, further studies could evaluate the relationship between the difference in ingested and expended calories with PA, and their joint effect on NAFLD.

## 6. Conclusions

Individuals with high PA showed higher energy expenditure than low PA peers. PA had positive influence on MetS parameters if fewer calories were ingested than those expended. High PA increased AST levels but had positive effects on ALT and GGT levels and IFC-NMR in NAFLD patients. It contributed to reinforce the evidence that the Mediterranean lifestyle, with Mediterranean diet and regular PA, improves MetS and NAFLD.

## Figures and Tables

**Table 1:** Sociodemographic characteristics according to physical activity levels.

	Low PA	High PA	
	Median (IQR)	Median (IQR)	*p* Value
Age (years)	56.0 (10.0)	52.0 (12.0)	<0.001
Anthropometrics			
Weight (Kg)	93.8 (15.6)	94.8 (19.2)	0.034
BMI (Kg/m^2^)	32.5 (4.4)	33.6 (5.5)	0.094
WC (cm)	111.1 (14.4)	112.6 (12.5)	0.466
HC (cm)	110.9 (9.6)	113.4 (14.2)	0.006
NC (cm)	42.6 (3.4)	40.7 (6.1)	0.002
Diet			
Carbohydrates (g/day)	248.4 (122.3)	236.9 (131.5)	0.693
Protein (g/day)	91.4 (36.6)	113.6 (48.3)	0.003
Lipids (g/day)	100.0 (42.4)	93.9 (43.1)	0.011
Ingested calories (Kcal/day)	2311.0 (960.6)	2321.2 (1013.6)	0.442
Expended calories (Kcal/day)	1700.2 (708.8)	2515.3 (856.8)	<0.001
Ingested–expended calories (Kcal/day)	642.5 (1256.3)	−143.9 (1174.4)	<0.001
Energy expenditure			
Measured accelerometer (MET/day)	1.8 (0.2)	2.1 (0.4)	<0.001
Reported Minnesota (MET/day)	0.3 (0.4)	0.4 (0.5)	<0.001
Measured-reported (MET/day)	1.5 (0.3)	1.9 (0.5)	<0.001
Education (years)	15.0 (4.0)	15.0 (6.0)	0.607
	n (%)	n (%)	
Gender			0.002
Female	12 (30.8)	19 (47.5)	
Socioeconomic status according job			0.068
Low	20 (62.5)	17 (77.3)	
Medium	10 (31.2)	4 (18.2)	
High	2 (6.2)	1 (4.5)	
Smoking habit ≥ 1 cigarette/day	2 (5.1)	7 (17.5)	0.001
Alcohol consumption ≥ 7 drinks/week	5 (12.8)	9 (22.5)	0.048

Abbreviations: BMI = body mass index, HC = hip circumference, MET = metabolic equivalents of task, NC = neck circumference, PA = physical activity, WC = waist circumference. Physical activity levels are classified according to weekly METs/day (high: >P50, low: <P50). Differences between medians low PA vs high PA were tested by Mann–Whitney U-test. Differences in prevalence’s across groups were examined using χ².

**Table 2:** Health characteristics according to physical activity levels.

	Low PA	High PA	
	Median (IQR)	Median (IQR)	*p* Value
Liver profile			
AST (U/L)	24.0 (12.5)	23.0 (8.0)	<0.001
ALT (U/L)	30.5 (29.5)	24.0 (18.0)	<0.001
GGT (U/L)	33.0 (28.5)	32.0 (25.0)	0.001
FLI	91.0 (10.0)	92.0 (10.7)	0.457
HSI	45.3 (6.0)	44.3 (7.9)	0.958
IFC-NMR (%)	12.3 (12.1)	10.3 (6.5)	<0.001
	n (%)	n (%)	
NAFLD according to			
AST > 40 U/L	2 (5.4)	3 (7.7)	0.421
ALT > 40 U/L	15 (38.5)	8 (20.0)	<0.001
GGT > 50 U/L men; > 32 U/L women	16 (41.0)	13 (32.5)	0.116
FLI ≥ 60	38 (100.0)	38 (95.0)	0.005
HSI > 36	35 (94.6)	38 (97.4)	0.203
IFC-NMR > 6.4%	33 (84.6)	30 (75.0)	0.033
Metabolic syndrome parameters			
Abdominal obesity (WC ≥ 94 cm men; ≥ 80 cm women)	37 (94.9)	39 (97.5)	0.222
High triglyceridemia ≥150 mg/dL	27 (69.2)	23 (57.5)	0.031
Low HDL-C <40 mg/dL men; <50 mg/dL women	23 (59.0)	25 (62.5)	0.521
Hypertension (systolic pressure ≥ 130 and/or diastolic pressure ≥ 85 mmHg)	30 (78.9)	26 (66.7)	0.016
High fasting glycemia ≥ 100 mg/dL	25 (64.1)	18 (45.0)	0.001

Abbreviations: ALT = alanine aminotransferase, AST = aspartate aminotransferase, FLI = fatty liver index, GGT = gamma glutamyl transferase, HSI = hepatic steatosis index, HDL-C = high density lipoprotein-cholesterol, IFC-NMR = intrahepatic fat content by nuclear magnetic resonance, NAFLD = non-alcoholic fatty liver disease, PA = physical activity. Physical activity levels are classified according to weekly METs/day (high: >P50, low: <P50). Differences between medians low PA vs high PA were tested by Mann–Whitney U-test. Differences in prevalence’s across groups were examined using χ².

**Table 3:** Association between metabolic syndrome parameters (dependent variables) and physical activity levels (independent variables).

		Low PA	High PA	
		OR (95% CI)	OR (95% CI)	*p*-Value
Abdominal obesity	Crude OR	1.00 (ref.)	2.11 (0.62–7.15)	0.231
	OR Adjusted 1	1.00 (ref.)	1.79 (0.48–6.68)	0.387
	OR Adjusted 2	1.00 (ref.)	1.42 (0.38–5.28)	0.601
High triglyceridemia	Crude OR	1.00 (ref.)	0.60 (0.38–0.96)	0.031
	OR Adjusted 1	1.00 (ref.)	0.48 (0.29–0.82)	0.007
	OR Adjusted 2	1.00 (ref.)	0.67 (0.37–1.19)	0.172
Low HDL-C	Crude OR	1.00 (ref.)	1.16 (0.74–1.82)	0.521
	OR Adjusted 1	1.00 (ref.)	0.80 (0.48–1.32)	0.374
	OR Adjusted 2	1.00 (ref.)	0.76 (0.44–1.31)	0.323
Hypertension	Crude OR	1.00 (ref.)	0.53 (0.32–0.89)	0.016
	OR Adjusted 1	1.00 (ref.)	0.75 (0.43–1.30)	0.305
	OR Adjusted 2	1.00 (ref.)	0.98 (0.52–1.85)	0.957
High fasting glycemia	Crude OR	1.00 (ref.)	0.46 (0.29–0.72)	0.001
	OR Adjusted 1	1.00 (ref.)	0.55 (0.33–0.91)	0.019
	OR Adjusted 2	1.00 (ref.)	0.68 (0.39–1.17)	0.161

Abbreviations: OR: odds ratio; ref.: reference. OR adjusted 1: odds ratio adjusted by sociodemographic characteristics (age and gender). OR Adjusted 2: odds ratio adjusted by sociodemographic characteristics (age, gender, and ingested–expended calories). HDL-C = high density lipoprotein-cholesterol, PA = physical activity. Physical activity levels are classified according to weekly METs/day (high: >P50, low: <P50).

**Table 4:** Association between indicators of NAFLD (dependent variables) and physical activity levels (independent variables).

		Low PA	High PA	
		OR (95% CI)	OR (95% CI)	*p*-Value
AST	Crude OR	1.00 (ref.)	1.46 (0.58–3.68)	0.424
	OR Adjusted 1	1.00 (ref.)	1.46 (0.56–3.78)	0.439
	OR Adjusted 2	1.00 (ref.)	4.14 (1.26–13.58)	0.019
	OR Adjusted 3	1.00 (ref.)	7.26 (1.79–29.40)	0.005
ALT	Crude OR	1.00 (ref.)	0.40 (0.24–0.66)	<0.001
	OR Adjusted 1	1.00 (ref.)	0.43 (0.26–0.73)	0.002
	OR Adjusted 2	1.00 (ref.)	0.24 (0.12–0.47)	<0.001
	OR Adjusted 3	1.00 (ref.)	0.24 (0.12–0.48)	<0.001
GGT	Crude OR	1.00 (ref.)	0.69 (0.44–1.10)	0.117
	OR Adjusted 1	1.00 (ref.)	0.76 (0.47–1.24)	0.272
	OR Adjusted 2	1.00 (ref.)	0.52 (0.29–0.94)	0.031
	OR Adjusted 3	1.00 (ref.)	0.57 (0.31–1.04)	0.066
HSI	Crude OR	1.00 (ref.)	2.17 (0.64–7.37)	0.214
	OR Adjusted 1	1.00 (ref.)	3.48 (0.95–12.80)	0.061
	OR Adjusted 2	1.00 (ref.)	1.06 (0.29–3.93)	0.927
	OR Adjusted 3	1.00 (ref.)	1.05 (0.15–7.15)	0.963
IFC-NMR	Crude OR	1.00 (ref.)	0.55 (0.31–0.96)	0.035
	OR Adjusted 1	1.00 (ref.)	0.78 (0.42–1.42)	0.412
	OR Adjusted 2	1.00 (ref.)	0.28 (0.14–0.55)	<0.001
	OR Adjusted 3	1.00 (ref.)	0.26 (0.12–0.56)	<0.001

Abbreviations: OR: odds ratio; ref.: reference. OR adjusted 1: odds ratio adjusted by health characteristics (elevated fasting glucose, elevated triglycerides, and hypertension). OR Adjusted 2: odds ratio adjusted by sociodemographic characteristics (age, gender, smoking habit, alcohol consumption, and ingested–expended calories). OR Adjusted 3: odds ratio adjusted by health and sociodemographic characteristics (elevated fasting glucose, elevated triglycerides, hypertension, age, gender, smoking habit, alcohol consumption, and ingested–expended calories). ALT = alanine aminotransferase, AST = aspartate aminotransferase, GGT = gamma-glutamyl transferase, HSI = hepatic steatosis index, IFC-NMR = intrahepatic fat content by nuclear magnetic resonance, NAFLD = non-alcoholic fatty liver disease, PA = physical activity. Physical activity levels are classified according to weekly METs/day (high: >P50, low: <P50).

## Data Availability

There are restrictions on the availability of data for this trial, due to the signed consent agreements around data sharing, which only allow access to external researchers for studies following the project’s purposes. Requestors wishing to access the trial data used in this study can make a request to pep.tur@uib.es.

## Clinical Trials Registration

The trial was registered at ClinicalTrials.gov with registry number NCT04442620 (https://clinicaltrials.gov/ct2/show/NCT04442620; accessed on 30 April 2021).

## Abbreviations

ALT	alanine aminotransaminase
AST	aspartate aminotransferase
BMI	body mass index
CI	confidence interval
FFQ	food frequency questionnaire
FLI	fatty liver index
GGT	gamma-glutamyl transferase
HDL-cholesterol	high density lipoprotein cholesterol
HSI	hepatic steatosis index
IFC-NMR	intrahepatic fat content by nuclear magnetic resonance
IQR	interquartile range
MET	metabolic equivalent task of task
MetS	metabolic syndrome
MRI	magnetic resonance imaging
NAFLD	non-alcoholic fatty liver disease
OR	odds ratio
PA	physical activity
P50	50th percentile
SD	standard deviations
